# Hypervalent hydridosilicate in the Na–Si–H system

**DOI:** 10.3389/fchem.2023.1251774

**Published:** 2023-09-08

**Authors:** Kristina Spektor, Holger Kohlmann, Dmitrii Druzhbin, Wilson A. Crichton, Shrikant Bhat, Sergei I. Simak, Olga Yu Vekilova, Ulrich Häussermann

**Affiliations:** ^1^ Inorganic Chemistry, Faculty for Chemistry and Mineralogy, Leipzig University, Leipzig, Germany; ^2^ Deutsches Elektronen-Synchrotron DESY, Hamburg, Germany; ^3^ ESRF-The European Synchrotron Radiation Facility, Grenoble, France; ^4^ Theoretical Physics Division, Department of Physics, Chemistry and Biology (IFM), Linköping University, Linköping, Sweden; ^5^ Department of Physics and Astronomy, Uppsala University, Uppsala, Sweden; ^6^ Department of Materials and Environmental Chemistry, Stockholm University, Stockholm, Sweden

**Keywords:** hydridosilicate, gigapascal hydrogenation, multi-anvil techniques, crystal structure prediction, hypervalency

## Abstract

Hydrogenation reactions at gigapascal pressures can yield hydrogen-rich materials with properties relating to superconductivity, ion conductivity, and hydrogen storage. Here, we investigated the ternary Na–Si–H system by computational structure prediction and *in situ* synchrotron diffraction studies of reaction mixtures NaH–Si–H_2_ at 5–10 GPa. Structure prediction indicated the existence of various hypervalent hydridosilicate phases with compositions Na_m_SiH_(4+m)_ (m = 1–3) at comparatively low pressures, 0–20 GPa. These ternary Na–Si–H phases share, as a common structural feature, octahedral SiH_6_^2−^ complexes which are condensed into chains for m = 1 and occur as isolated species for m = 2, 3. *In situ* studies demonstrated the formation of the double salt Na_3_[SiH_6_]H (Na_3_SiH_7_, m = 3) containing both octahedral SiH_6_^2−^ moieties and hydridic H^−^. Upon formation at elevated temperatures (>500°C), Na_3_SiH_7_ attains a tetragonal structure (*P*4/*mbm*, *Z* = 2) which, during cooling, transforms to an orthorhombic polymorph (*Pbam*, *Z* = 4). Upon decompression, *Pbam*-Na_3_SiH_7_ was retained to approx. 4.5 GPa, below which a further transition into a yet unknown polymorph occurred. Na_3_SiH_7_ is a new representative of yet elusive hydridosilicate compounds. Its double salt nature and polymorphism are strongly reminiscent of fluorosilicates and germanates.

## Graphical Abstract

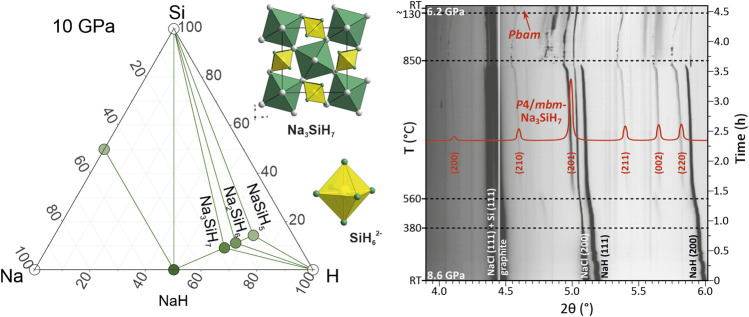

## 1 Introduction

The alkali metal A–Si–H systems (A = Li, Na, and K) have recently attracted attention because computational predictions suggested the existence of hydrogen-rich ternary phases at high pressures with potentially superconducting, superionic, and/or hydrogen storage properties (Liang et al., 2020; Zhang et al., 2020; Liang et al., 2021; Wu et al., 2022; Xie et al., 2022). According to these predictions, at lower pressures (up to 50 GPa), A–Si–H systems commonly possess a stable compound, A_2_SiH_6_, featuring octahedral SiH_6_^2−^ species in which Si attains a hypervalent bonding situation. Na_2_SiH_6_ was suggested being a H^−^ superionic conductor in the pressure range 4–10 GPa (Liang et al., 2021) and at temperatures around 1,000°C, and K_2_SiH_6_ has been attributed favorable H-storage properties (Xie et al., 2022).

At higher pressure (50–200 GPa), more varied compositions become stable, with structures where octahedral units are connected and/or H_2_ molecules are additionally incorporated (i.e., KSiH_7_, KSiH_8_, K_2_SiH_8_, Na_2_SiH_14_, and Na_3_SiH_10_) (Liang et al., 2021; Xie et al., 2022). For A = Li, the structures of the predicted phases deviate frequently from the octahedral theme (Liang et al., 2020; Zhang et al., 2020). Also, for Li–Si–H, there seems to be a greater variety of stable hydrogen-rich structures and compositions at high pressures (e.g., LiSiH_5_, LiSiH_6_, Li_3_SiH_10_, Li_2_SiH_10_, and Li_2_SiH_12_). LiSi_2_H_9_ and LiSiH_8_ were predicted to become good phonon-mediated superconductors at pressures above 170 GPa (Liang et al., 2020).

Despite the interesting results from computational structure prediction, hitherto only polymorphic K_2_SiH_6_ has been reported from experimental high-pressure investigations (Puhakainen et al., 2012; Vekilova et al., 2023). This compound is stable even at ambient pressure where it adopts the cubic K_2_PtCl_6_ structure. At pressures above 8 GPa, K_2_SiH_6_ crystallizes in a trigonal structure (Vekilova et al., 2023).

Here, we report on the re-examination of the Na–Si–H system by crystal structure prediction and *in situ* studies of reactions m NaH + Si + 4 H_2_ (m = 1, 2) at pressures up to 10 GPa. For this, we employed a large-volume press (LVP) high pressure methodology which provides well-controlled *p, T* environments for high pressure hydrogenation reactions (Spektor et al., 2020a; Spektor et al., 2020b). Our initial intention was to obtain the predicted (superionic) compound Na_2_SiH_6_ with a simple *P*¯3*m*1 structure (Liang et al., 2021), which is also the structure of the high pressure polymorph of K_2_SiH_6_ (Vekilova et al., 2023). However, in contrast with earlier reports, we find evidence for the existence of multiple Na–Si–H phases along the composition line Na_m_SiH_(4+m)_ (m = 1, 2, 3) at comparatively low pressures up to 20 GPa, among which polymorphic Na_3_SiH_7_ was experimentally observed.

## 2 Materials and methods

### 2.1 High pressure experiments and data analysis

All steps of sample preparation and recovery were performed in a glove box under argon atmosphere. Powdered NaH (Sigma-Aldrich, 90%) and powdered Si [325 mesh, 99.999% (metals basis), Thermo Scientific] were mixed at a molar ratio of 1:1 and 2:1 (NaH:Si) and compressed into pellets with an outer diameter (OD) of 2 mm. Ammonia borane (BH_3_NH_3_, Sigma-Aldrich, 90%) served as a hydrogen source since it has a well-defined decomposition behavior at high pressures and produces chemically inert BN as a residue (Nylén et al., 2009). The amount of BH_3_NH_3_ used for each sample corresponded to an approx. 4× molar excess of H_2_ with respect to Si. NaH/Si sample pellets were sandwiched between pelletized BH_3_NH_3_ and sealed inside NaCl capsules with 3.0 mm OD.

*In situ* synchrotron diffraction high-pressure experiments were performed at beamline ID06-LVP at the ESRF and employed 14/7 multi-anvil assemblies, which are described in detail elsewhere (Vekilova et al., 2023). Amorphous SiBCN rods and either MgO or amorphous BCN epoxy were used as X-ray windows in the octahedra and gaskets, respectively, along the beam direction. Assemblies were compressed to target pressures ≈5 and ≈9 GPa and heated in a Voggenreiter-built modified-cubic press (Guignard and Crichton, 2015). Pressure was estimated *in situ* from PXRD diffraction patterns using the equation of state of NaCl by Matsui et al. (2012). The temperature was evaluated from power–*T* calibration curves. Angle-dispersive powder X-ray diffraction patterns were collected in the 1.27°–15.26° 2θ range at a constant wavelength (*λ* = 0.233933 Å). Data were acquired using the Pilatus3X-900 kW CdTe high-resolution 2D detector. The *in situ* data were integrated, visualized, and manipulated using Fit2D software (Hammersley, 2016). Indexing of the powder patterns was performed using DICVOL and TAUP algorithms within the CRYSFIRE package (Shirley, 2004). Le Bail fitting (Le Bail et al., 1988) and Rietveld refinement (Rietveld, 1969) against the *in situ* data were performed in Jana 2006 (Petříček et al., 2014). A detailed description of the high-pressure experiments and data analysis is given in Supplementary Material.

### 2.2 Theoretical calculations

The Na–Si–H system was studied with a crystal structure prediction methodology using Ab initio random structure searching implemented in the code AIRSS (Pickard and Needs, 2006; Pickard and Needs, 2011) and an evolutionary algorithm implemented in the Universal Structure Predictor: Evolutionary Xtallography (USPEX) code (Oganov and Glass, 2006; Oganov et al., 2011; Lyakhov et al., 2013), both coupled with the Vienna Ab Initio Simulation Package (VASP) (Kresse and Hafner, 1993; Kresse and Furthmüller, 1996). VASP calculations were based on a first-principles projector-augmented wave (PAW) method (Blöchl, 1994) within the density functional theory (DFT) (Hohenberg and Kohn, 1964; Kohn and Sham, 1965). The generalized gradient approximation for exchange and correlation potential and energy was used in its Perdew–Burke–Erzernhof (PBE) (Perdew et al., 1996; Perdew et al., 1997) flavor. H at zero pressure was calculated as a molecule (H_2_) and at higher pressures as a solid with an *I*4/*mmm* structure (according to the work of Pickard and Needs (2007)).

All Na_m_SiH_(4+m)_ (m = 1, 2, 3) model structures were fully relaxed; i.e., volume, shape, and internal atomic positions were adjusted to get nearly zero forces and stresses (stress components of order 0.1 GPa and forces of order 0.01 eV/Å maximum). The considered pressures were 0, 10, and 20 GPa. The Monkhorst–Pack (Monkhorst and Pack, 1976) k-point density for integrations over the Brillouin zone was set with 0.2. The energy cutoff for plane waves was set to 320 eV. All the static calculations were conducted at temperature T = 0 K.

Phonons were calculated from the force constants in real space obtained by VASP using density functional perturbation theory (DFPT) used as an input for the PHONOPY program (Togo and Tanaka, 2015). A plane-wave energy cutoff of 500 eV was used in all the corresponding calculations. A 2 × 2 × 2 supercell of each structure was used. To investigate the dynamical stability of *P*4/*mbm* Na_3_SiH_7_ at experimentally relevant temperature of 600 K and pressure 10 GPa, *ab initio* molecular dynamics (AIMD) calculations, as implemented in VASP, were conducted and then post-processed by the temperature-dependent effective potential method (TDEP) (Hellman et al., 2011; Hellman et al., 2013). TDEP maps the AIMD data onto a model Hamiltonian and provides the best harmonic fit to the system of anharmonic vibrating atoms at a particular temperature. Therefore, effective temperature-dependent phonon dispersions are obtained, and in particular, dynamical stability due to anharmonic vibrations can be studied. AIMD simulation were performed in 2 × 2 × 2 supercells (176 atoms) for *P*4*/mbm* Na_3_SiH_7_, with 2 × 2 × 2 k points, and 500 eV energy cutoff for the plane waves, with the same PAW potentials and exchange-correlation as in the static calculations. The canonical NVT ensemble using a Nosé–Hoover thermostat with the default Nosé mass as set by VASP and a 0.5 fs time step was applied. The data from 600 time steps after equilibration were used for the extraction of temperature-dependent force constants.

## 3 Results and discussion

### 3.1 Revisiting structure prediction for the Na–Si–H system

A recent exploration of the Na–Si–H system for stable ternary high-pressure phases yielded only one compound, trigonal *P*¯3*m*1 Na_2_SiH_6_, up to 50 GPa (Liang et al., 2021). At pressures below 3 GPa, the proposed Na_2_SiH_6_ is not dynamically stable, i.e., its phonon dispersion has branches with imaginary frequencies. This indicates lattice instability, and as a consequence, there should be a more stable structure with this composition, or decomposition into a different composition. We re-examined structure predictions (and also introduced a number of trial structures from chemical intuition) allowing for *Z* = 4 (*Z* = number of formula units) and focused on the low-pressure region up to 20 GPa which would be accessible in LVP hydrogenations. The dominant feature of SiH_6_^2−^ octahedral units in low-pressure structures, as established from previous A–Si–H structure prediction work (Liang et al., 2020; Liang et al., 2021; Zhang et al., 2020; Wu et al., 2022; Xie et al., 2022), together with chemical reasoning, suggested that compositions Na_m_SiH_(4+m)_ (m = 1, 2, 3) are most plausible, and we restricted our search to these compositions. Phase diagrams were established for 0, 10, and 20 GPa. Compared to earlier work, a radically different picture evolved.

**FIGURE 1:**
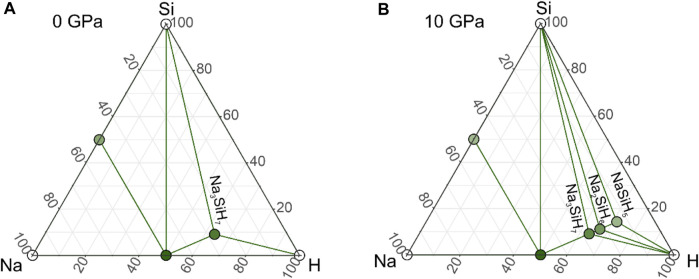
Na–Si–H phase diagram at **(A)** 1 atm and **(B)** 10 GPa. Large green circles represent compounds along the composition line mNaH + SiH_4_ (m = 1, 2, 3) which are located on the convex hull. Note that Na_3_SiH_7_ in the here-established *Pbam* structure is not dynamically stable at 0 GPa.

**FIGURE 2:**
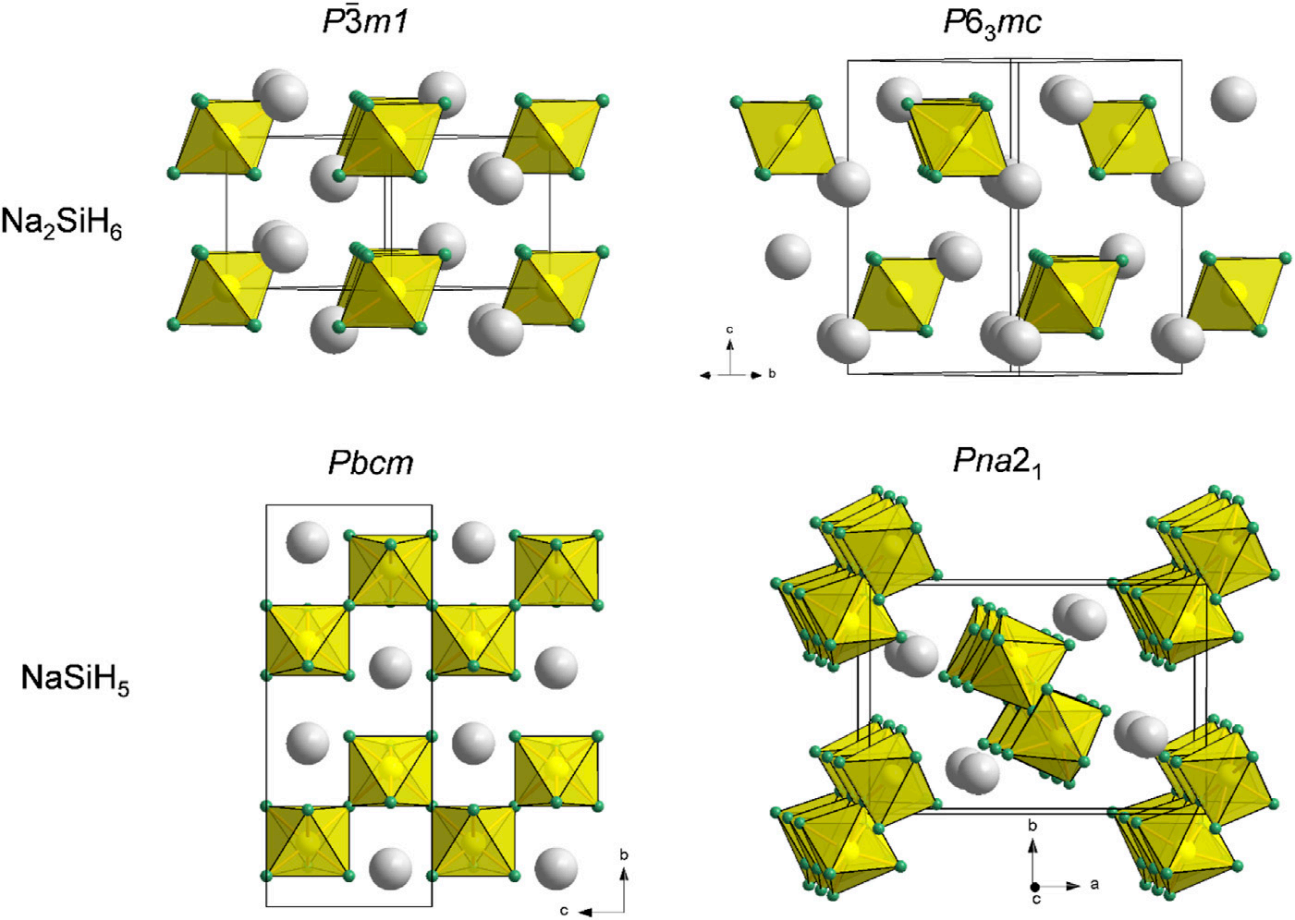
Compilation of predicted structures for Na_m_SiH_(4+m)_ (m = 1, 2) compositions. Na, Si, and H atoms are depicted as gray, yellow, and green circles, respectively. SiH_6_^2−^ octahedra are shown in yellow.

Figure 1 shows the phase diagrams for ambient pressure and 10 GPa (the phase diagram for 20 GPa is virtually identical to that for 10 GPa). In Supplementary Figure S5, phonon dispersions of some of the predicted structures are depicted. At ambient pressure (Figure 1A), a compound Na_3_SiH_7_ with an orthorhombic *Pbam* structure appeared to be stable with respect to decomposition into NaH, NaSi, Si, and H_2_ but was not dynamically stable. This suggests that the composition Na_3_SiH_7_ is stable at ambient pressure but should occur in a different, most likely more complex, structure. As a matter of fact, identifying Na_3_SiH_7_ as a potential stable composition at ambient pressure was rather inferred from the experimental results than initially obtained from computational structure prediction. We will discuss the structures for Na_3_SiH_7_ and their stability in detail later. Furthermore, at ambient pressure, we found a hexagonal form of Na_2_SiH_6_ with *P*6_3_ *mc* symmetry slightly (by ≈0.05 eV/Z) more stable than *P*¯3*m*1-Na_2_SiH_6_. However, the enthalpy of Na_2_SiH_6_ is still above the convex hull, and both polymorphs are not dynamically stable at ambient pressure (cf. Supplementary Figures 5D, E). The Na_2_SiH_6_ structures are depicted in Figure 2.

At 10 GPa, we find that the complete sequence of compositions Na_m_SiH_(4+m)_ (m = 1, 2, 3) is stable, both thermodynamically and dynamically. *Pbam*-Na_3_SiH_7_, which was found only enthalpically stable at ambient pressure, is now also dynamically stable. With pressure, the trigonal Na_2_SiH_6_ polymorph stabilizes over the hexagonal one and also becomes dynamically stable. NaSiH_5_ structures are not expected to realize separated SiH_6_^2−^ octahedral units but chains of corner-condensed or dimers of edge-condensed octahedra, [SiH_4_H_2/2_^−^]_∞_ and Si_2_H_10_^2−^, respectively. Indeed, *Pbcm*-NaSiH_5_, which is isostructural to BaAlH_5_ (Figure 2) (Zhang et al., 2002), was found on the convex hull. At the same time, a different octahedral chain structure, *Pna*2_1_-NaSiH_5_ [isostructural to SrAlH_5_ (Sato et al., 2018)], is only about 0.15 eV/Z less stable (Figure 2). It can be speculated that the composition NaSiH_5_ gives rise to a manifold of enthalpically close-lying polymorphic structures (Weidenthaler et al., 2006; Shlyapnikov et al., 2018) and that, most likely, the most stable one has yet to be identified. Supplementary Tables S3–S5 in Supplementary Material list the parameters of some Na_m_SiH_(4+m)_ structures. It is also easy to understand that computational structure prediction meets severe difficulties when tackling ternary compositions with complex structural features (Kvashnin et al., 2020).

### 3.2 *In situ* experiments and identification of Na_3_SiH_7_

Reactions m NaH + Si + 4 H_2_ (m = 1, 2), targeting originally proposed Na_2_SiH_6_, were performed at around 5 and 9 GPa. After compressing to target pressure, the reaction mixtures were initially heated to a temperature around 400°C at which the H-source BH_3_NH_3_ is expected to be completely decomposed into h-BN and hydrogen fluid (Nylén et al., 2009). The samples were then equilibrated for about 15–20 min. The reaction mixtures behaved very similar, independent of starting composition (m = 1 or 2) or pressure (5 or 9 GPa). In the following, reported results refer to m = 2 (2NaH:1Si) mixtures.

**FIGURE 3:**
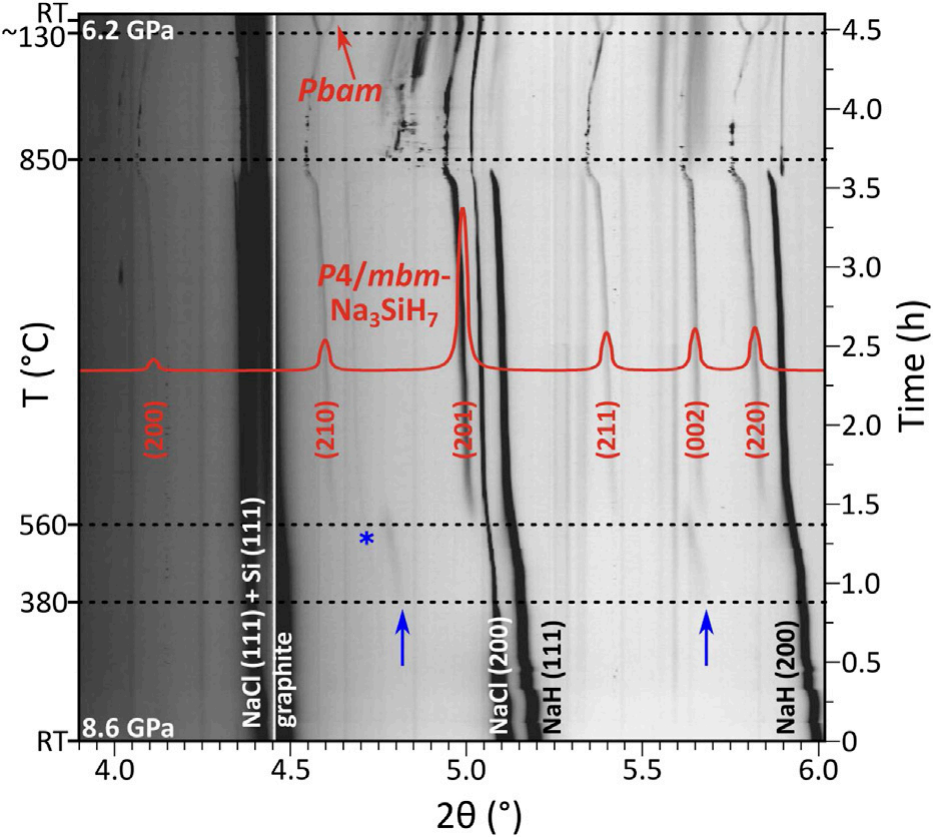
Compilation of PXRD patterns (*λ* = 0.233933 Å) acquired during the *p* ≈ 9 GPa experiment (2NaH:1Si starting mixture) (observed intensities are shown on the logarithmic scale). Overlain is the simulated pattern of *P*4/*mbm*-Na_3_SiH_7_ (shown in red, linear scale) based on the DFT optimized model structure (Supplementary Table S5D). Blue arrows mark the appearance of an intermediate hexagonal phase (*a* ≈ 4.75 Å, *c* ≈ 7.65 Å). The blue asterisk marks a peak belonging to an additional unidentified phase, growing in parallel with Na_3_SiH_7_ and disappearing upon heating above ≈830°C. Splitting of the tetragonal reflections upon transition to the *Pbam* phase is visible on cooling below 130°C (at the very top of the figure).

Figure 3 shows the evolution of diffraction patterns for the *p* ≈ 9 GPa run. In this experiment, one can recognize the growth of an intermediate phase above ≈380°C, which manifests as broad low-intensity diffraction peaks. The peaks could be indexed to a hexagonal unit cell (*a* ≈ 4.75 Å, *c* ≈ 7.65 Å), yet this phase remains uncharacterized due to the diffuse character and poor intensity of its reflections. Above ≈560°C, the peaks of the intermediate phase are superseded by another set of reflections, which were indexed to a primitive tetragonal unit cell with *a* ≈ 6.59 and *c* ≈ 4.78 Å (at ≈7.2 GPa and 770°C, as observed in the *p* ≈ 5 GPa run). *P*4/*mbm* was derived as the highest applicable space group from the extinction symbol *P*–*b*–. The symmetry and lattice parameter ratio (*c*/*a* ≈ 0.725) suggested an isostructural relation to K_3_SiF_7_ (*c*/*a* = 0.719 at ambient pressure) (Deadmore and Bradley, 1962; Hofmann and Hoppe, 1979) and, thus, a composition Na_3_SiH_7_. Calculated reflection intensities for K_3_SiF_7_-type Na_3_SiH_7_ matched closely with the experiment. Furthermore, with the knowledge of composition and formula units, *P*4/*mbm*-Na_3_SiH_7_ could be obtained as a low-enthalpy phase from the computational structure prediction methodology. The DFT optimized structure (Supplementary Tables S5D, E) was then used in Rietveld analysis of the *in situ* PXRD data (≈7.2 GPa, 770°C), yielding a reasonable fit (*R*_obs_≈5.5%) despite the low phase fraction of Na_3_SiH_7_ (Supplementary Figure S3). Details of the refinement process as well as the corresponding plot and extracted structural data are provided in Supplementary Material (Supplementary Tables S1, S2).

The formation of *P*4/*mbm*-Na_3_SiH_7_ during hydrogenations of NaH–Si mixtures requires pressures above 5 GPa (cf. Supplementary Figures S2, S4). In the *p* ≈ 5 GPa hydrogenation experiment, the intermediate hexagonal phase was not seen. Diffraction peaks from *P*4/*mbm*-Na_3_SiH_7_ become noticeable above ≈490°C. Irrespective of pressure, the *P*4/*mbm*-Na_3_SiH_7_ phase grows very sluggishly even when raising temperatures up to 850°C. This indicates a rather high thermal stability of *P*4/*mbm*-Na_3_SiH_7_ at high-pressure conditions and shows at the same time that elemental Si represents a rather unreactive precursor.

**FIGURE 4:**
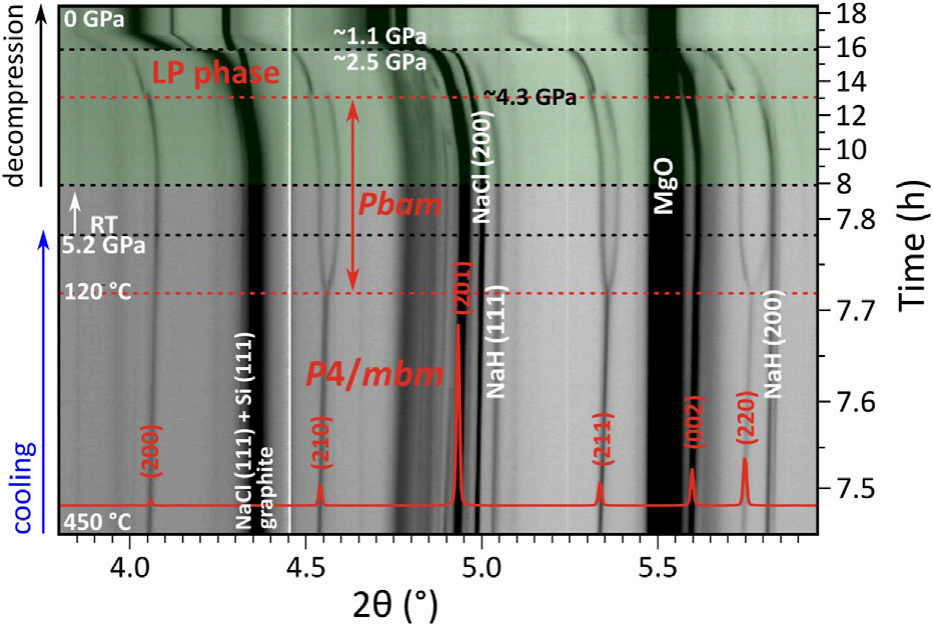
Compilation of PXRD patterns (*λ* = 0.233933 Å) acquired during the *p* ≈ 5 GPa experiment (2NaH:1Si starting mixture) during cooling and decompression. Observed intensities are shown on the logarithmic scale. Data collected upon decompression (1 pattern/min) are highlighted in green. The transition of *P*4/*mbm*-Na_3_SiH_7_ to a *Pbam* polymorph occurs below 120°C, and a further transition to an unidentified low-pressure polymorph is visible below ≈4.3 GPa (both events are marked with red dashed lines). Overlain is the simulated pattern of *P*4/*mbm*-Na_3_SiH_7_ (shown in red, linear scale) based on the refined structure model (Supplementary Tables S1, S2). Mismatch between observed and calculated intensities is due to texture which developed in Na_3_SiH_7_ above ≈770°C (cf. Supplementary Figure S4).

Upon cooling from (high) synthesis temperatures to below 120°C, the tetragonal diffraction pattern showed a clear splitting into an orthorhombic one, which appeared continuous and was accompanied with a doubling of unit cell (*a*_o_ ≈ *b*_o_ = √2*a*_t_, *c*_o_ ≈ *c*_t_). For the *p* ≈ 9 GPa run, this feature is included in Figure 3. For the *p* ≈ 5 GPa experiment, this tetragonal-to-orthorhombic transition is shown in Figure 4. The reflections were indexed to a unit cell *a* ≈ 9.23 Å, *b* ≈ 9.36 Å, and *c* ≈ 4.76 Å (at *p* ≈ 5.2 GPa, ≈RT). Again, suspecting analogy with fluorides, the (NH_4_)_3_GeF_7_ structure (*Pbam* space group symmetry, Z = 4) (Mel’nikova et al., 2016; Mel’nikova et al., 2017; Bogdanov et al., 2019) was assigned to the orthorhombic phase.

In both 5 and 9 GPa runs, a further transition was noticeable upon decompression below ≈4.5 GPa, see again Figure 4 (and Supplementary Material for details). Indexing of the diffraction peaks arising at ≈4 GPa suggested as best-fitting candidates two primitive tetragonal unit cells (parameters are given relative to the initial tetragonal phase): a) *a* ≈ √2*a*_t_, *c* ≈ 2*c*_t_ (*a* ≈ 9.42 Å, *c* ≈ 9.61 Å, *Z* = 8); b) *a* ≈ *a*_t_, *c* ≈ 2*c*_t_ (*a* ≈ 6.66 Å, *c* ≈ 9.61 Å, *Z* = 4). The structure was difficult to resolve due to peak overlap as well as the occurrence of sudden pressure drops during decompression which obscured analysis. Further structure prediction work would be required to establish the structure and corresponding space group. Also, it remains unclear whether Na_3_SiH_7_ is recoverable to ambient pressure. The *ex situ* analysis of the only partially reacted run products was inconclusive.

### 3.3 Phase relations in Na_3_SiH_7_

**FIGURE 5:**
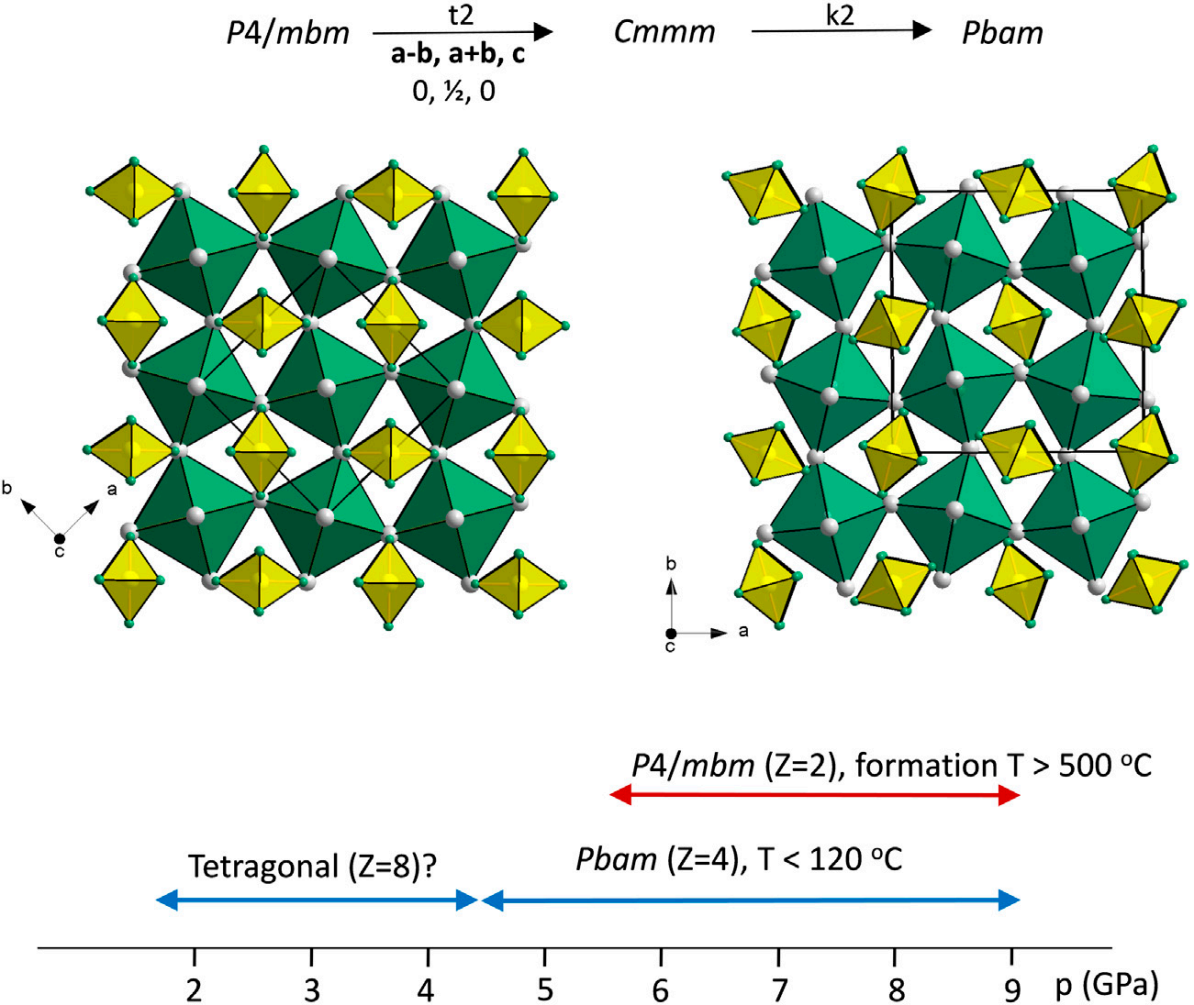
Structures of *P*4/*mbm*-Na_3_SiH_7_ (left) and *Pbam*-Na_3_SiH_7_ (right). Their group–subgroup relation is depicted at the top, and the experimentally observed pressure stability ranges are shown at the bottom. Na, Si, and H atoms are shown as gray, yellow, and green circles, respectively. SiH_6_^2−^ and HNa_6_ octahedra are shown in yellow and green, respectively.

Figure 5 shows the structures of polymorphic Na_3_SiH_7_ along with the *p, T* conditions for the observed phases. As mentioned, Na_3_SiH_7_ represents a double salt Na_3_[SiH_6_]H containing both octahedral SiH_6_^2−^ moieties and hydridic H. Hydridic H is octahedrally coordinated by six Na atoms, and HNa_6_ octahedra build up a corner-connected framework similar to the BO_3_ framework in perovskites ABO_3_. SiH_6_^2−^ octahedra are located in the voids accommodating the (larger-sized) A constituent of perovskites. Thus, the Na_3_SiH_7_ structures may be considered as an anti-perovskite arrangement (SiH_6_^2−^)[HNa_3_]^2+^, and as for perovskites, there is inherent structural flexibility from rotations and tilts of octahedra. *Pbam* is a subgroup of *P*4/*mbm*. The group-subgroup relationship is indicated in Figure 5. Analogous and also more extended sequences of phase transitions have been reported for fluorosilicate and fluorogermanate double salts [e.g., (NH_4_)_3_SiF_7_ and (NH_4_)_3_GeF_7_] as a function of temperature (Mel’nikova et al., 2014; Molokeev et al., 2014; Pogoreltsev et al., 2014; Mel’nikova et al., 2016; Mel’nikova et al., 2017; Bogdanov et al., 2019). These may potentially also include the yet unknown Na_3_SiH_7_ structure at room temperature and low pressures.

As previously mentioned, *Pbam*-Na_3_SiH_7_ was found enthalpically stable but dynamically unstable at ambient pressure. According to total energy calculations, this polymorph is by 0.4 eV/*Z* more stable than the *P*4/*mbm* structure. At 10 GPa, *Pbam*-Na_3_SiH_7_ is by about 0.24 eV more stable than the tetragonal form and dynamical stable, whereas *P*4/*mbm*-Na_3_SiH_7_ appears to be dynamically unstable at all pressures (cf. Supplementary Figures S5G, H).

**FIGURE 6:**
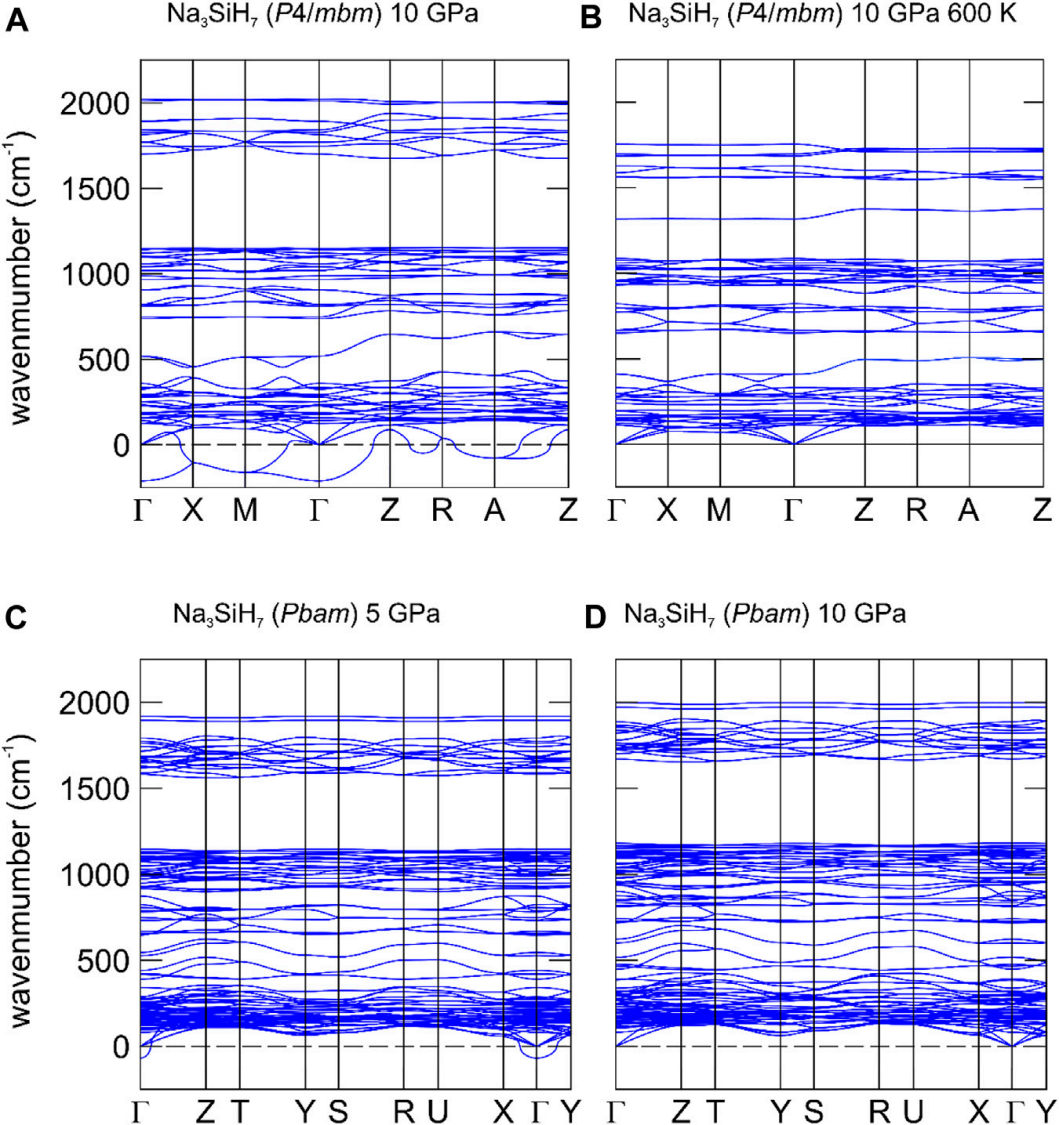
Phonon dispersion relations for *P*4*/mbm* and *Pbam* Na_3_SiH_7_ at different *p*, *T* conditions. **(A)** *P*4*/mbm*-Na_3_SiH_7_ at 10 GPa and 0 K. **(B)** *P*4*/mbm*-Na_3_SiH_7_ at 10 GPa and 600 K. **(C)** *Pbam*-Na_3_SiH_7_ at 5 GPa and 0 K. **(D)** *Pbam*-Na_3_SiH_7_ at 10 GPa and 0 K.

To investigate the dynamical stability of the *P*4/*mbm*-Na_3_SiH_7_ phase at experimentally relevant conditions, phonon dispersions were calculated in the framework of the TDEP method (Hellman et al., 2011; Hellman et al., 2013) at 10 GPa and 600 K. Figures 6A, B compare the phonon dispersions for 10 GPa at zero and 600 K. Si–H stretching and bending modes of the entities SiH_6_^2−^ (i.e., internal modes) and the (translational) modes of the hydridic H within Na_6_ octahedra are above around 500 cm^−1^, whereas SiH_6_^2−^ libration modes (i.e., rotation of octahedral units against each other) and optic translation modes of Na^+^ and SiH_6_^2−^ units (i.e., external modes) are below 500 cm^−1^.

At 0 K, *P*4/*mbm*-Na_3_SiH_7_ has two imaginary branches (Figure 6A). These originate from libration and acoustic modes which, according to the atom-decomposed phonon density of states (shown in Supplementary Figure S6), involve Na1 atoms (which are situated on the 4-fold axes 0,0,*z* and ½,½,*z*, cf. Figure 5) and the H atoms being part of SiH_6_ octahedra. The imaginary modes stabilize with temperature, which is attributed to the anharmonicity of atomic vibrations, causing renormalizing of the phonon modes due to phonon–phonon interaction (Figure 6B). Thus, *P*4/*mbm*-Na_3_SiH_7_ is stable at high-pressure and high-temperature conditions. Conversely, lowering temperature introduces dynamical instability, driving the distortion to the orthorhombic *Pbam* structure (*a*_o_ ≈ √2*a*_t_, *b*_o_ ≈ √2*a*_t_) which is dynamically stable at 10 GPa (Figure 6D). Reducing pressure to about 5 GPa introduces dynamical instability for the *Pbam* polymorph (Figure 6C), indicating a transition to yet another polymorph.

Thus, total energy and phonon calculations support the experimental findings described in the previous section. The structural chemistry and polymorphism of Na_3_SiH_7_ reminds of fluorosilicates and germanates A_3_Si/GeF_7_ (A = K, Rb, Cs, NH_4_). These materials have wide band gaps in the UV region (≈6 eV) in combination with interesting birefringent optical properties (Mel’nikova et al., 2016; Huang et al., 2020). In contrast, the calculated band structure of Na_3_SiH_7_ (Supplementary Figure S7) suggests a semiconductor with a direct band gap of around 2 eV. An interesting aspect, which has not been investigated in the course of this work, is a potentially superionic behavior of Na_3_SiH_7_. Superionicity of H^−^ was proposed in (yet) hypothetical Na_2_SiH_6_ where extraordinary diffusive H seems to be promoted by the hypervalent nature of SiH_6_^2−^ units (Liang et al., 2021). Against this background, it appears worthwhile to study the dynamical properties of Na_3_SiH_7_ over a wider temperature range by dedicated AIMD simulations.

## 4 Conclusion

*In situ* studies of reactions m NaH + Si + 2.5 H_2_ (m = 1, 2) at pressures up to 10 GPa revealed a new hypervalent hydridosilicate Na_3_SiH_7_ which corresponds to a double salt Na_3_[SiH_6_]H, featuring isolated octahedral SiH_6_^2−^ complexes and H^−^ anions. In the pressure range 5–10 GPa, Na_3_SiH_7_ occurs in a tetragonal high-temperature and orthorhombic low-temperature form. It is not yet clear whether and in which polymorphic form Na_3_SiH_7_ is recoverable to ambient pressure. Computation suggests the accessibility of further sodium hydridosilicate along the composition line Na_m_SiH_(4+m)_ (m = 1–3), i.e., Na_2_SiH_6_ and NaSiH_5_. The structures of phases Na_m_SiH_(4+m)_ constitute octahedral SiH_6_^2−^ complexes which are condensed for m = 1 and occur as isolated species for m = 2, 3. Interestingly, previously predicted superionic Na_2_SiH_6_ with a *P*¯3*m*1 structure has not been observed during our investigations. It may be speculated that this composition disappears from the convex hull once the most stable structure for NaSiH_5_ has been identified. The same may hold true for Na_3_SiH_7_ at ambient pressure. The composition NaSiH_5_ is expected to yield a manifold of enthalpically close-lying polymorphic structures based on corner- and edge-condensed SiH_6_^2−^ octahedra. In order to access NaSiH_5_ structures experimentally, the rather inert elemental Si should be replaced with a more active reactant, such as the Zintl phase NaSi. In addition to a higher reactivity, calculated reaction enthalpies with respect to NaSi (i.e., NaSi + 5/2H_2_) are about 0.4 eV/*Z* lower than with respect to NaH + Si + 2H_2_.

## Data Availability

The raw data supporting the conclusion of this article will be made available by the authors, without undue reservation.
