# Supplementary material for: Hypervalent hydridosilicate in the Na–Si–H system
Source: Front Chem. 2023 Sep 8;11:1251774. doi: 10.3389/fchem.2023.1251774 (PMC10515085; doi:10.3389/fchem.2023.1251774)
Supplement: Supplementary file 1 [file Presentation1.pdf]

SUPPORTING INFORMATION

Hypervalent hydridosilicates in the Na–Si–H system

Kristina Spektor,[1,2*] Holger Kohlmann,[1] Dmitrii Druzhbin,[3] Wilson A. Crichton,[3] Shrikant Bhat[2], Sergei I. Simak,[4,5] Olga Yu. Vekilova[6] and Ulrich Häussermann[6,*]

[1] *Inorganic Chemistry, Faculty for Chemistry and Mineralogy, Leipzig University, Johannisallee 29, 04103 Leipzig, Germany*

[2]*Deutsches Elektronen-Synchrotron DESY, Notkestraße 85, D-22607 Hamburg, Germany*

[3]*ESRF, The European Synchrotron Radiation Facility, F-38000 Grenoble, France*

[4]*Theoretical Physics Division, Department of Physics, Chemistry and Biology (IFM) Linköping University, SE-581 83, Linköping, Sweden*

[5]*Department of Physics and Astronomy, Uppsala University, SE-75120 Uppsala, Sweden*

[6]*Department of Materials and Environmental Chemistry, Stockholm University, SE-10691 Stockholm, Sweden*

Parts

I. High pressure experiments

Detailed description of experiments and data analysis

Figure S1. Temperature variations as a function of time for the NaH–Si–H_2 system at ≈5 GPa.

Figure S2. Temperature variations as a function of time for the NaH–Si–H_2 system at ≈9 GPa.

Figure S3. Results of the Rietveld fit of *P*4/*mbm*-Na_3SiH_7 model to *in situ* PXRD data at ≈770 °C, ≈7.2 GPa (5 GPa run).

Figure S4. Formation of *P*4/*mbm*-Na_3SiH_7 in 5 GPa run.

Table S1. Rietveld refinement results for *P*4/*mbm*-Na_3SiH_7 structure at ≈7.2 GPa, ≈770 °C.

Table S2. Fractional coordinates and atomic displacement parameters obtained for the *P*4/*mbm*-Na_3SiH_7 at ≈7.2 GPa, ≈770 °C.

II. Theoretical calculations.

Figure S5. Phonon dispersions for $Na_mSiH_{(4+m)}$ compounds at 0 and 10 GPa (and zero Kelvin).

Figure S6. Atom-projected phonon-density of states for *P*4/*mbm* Na_3SiH_7 at 10 GPa and zero Kelvin.

Figure S7. Electronic band structure and density of states of *P*4/*mbm*-Na_3SiH_7 at ambient pressure.

Table S3. Relaxed structures for $Pna2_1$ and *Pbcm* $NaSiH_5$ at various pressures

Table S4. Relaxed structures for $P6_3mc$ and $P\overline{3}m1$ Na_2SiH_6 at various pressures

Table S5. Relaxed structures for *P*4/*mbm* and *Pbam* Na_3SiH_7 at various pressures

I. High pressure experiments

1. Detailed description of experiments and data analysis

All steps of sample preparation were performed in a glove box under argon atmosphere. Powdered NaH (Sigma Aldrich, 90%) and powdered Si (325 mesh, 99.999% (metals basis)), Thermo Scientific) were carefully mixed at a molar ratio of 1:1 and 2:1 (NaH:Si) and compressed into pellets with an outer diameter (OD) of 2 mm and 0.9 – 1.1 mm height. Ammonia borane (BH_3NH_3, Sigma Aldrich, 90%) was used as hydrogen source since it has a well-defined decomposition behavior at high pressures and produces chemically inert BN as residual (Nylén et al., 2009). The amount of BH_3NH_3 used for each sample corresponded to a approx. 4× molar excess of H_2 with respect to Si. NaH/Si sample pellets were sandwiched between pelletized BH_3NH_3 and sealed inside NaCl capsules. The salt capsules had 3.0 mm OD and ~3.6 mm height.

High pressure experiments were performed at starting pressures of approx. 5 and 9 GPa and employed 14/7 multianvil assemblies. A detailed description of the 14/7 setup is provided elsewhere (Vekilova et al., 2023). In addition, 2 mm OD amorphous SiBCN rods and ~5.5 mm wide rectangles made of either MgO or amorphous BCN epoxy (TU Freiberg) were used as X-ray windows in the octahedra and gaskets, respectively, along the beam direction. Filled MgO octahedra were positioned between eight truncated tungsten carbide cubes (32 mm, grade TF09, Fujilloy Co. Ltd.) fitted with pyrophyllite gaskets. Assemblies were compressed at a rate of 1 bar/min oil pressure (~3.2–3.7 GPa/h) to the target pressures and heated in the Voggenreiter-built modified-cubic press at beamline ID06-LVP, ESRF (Guignard and Crichton, 2015). The heating was performed at various rates, ranging from ~10–20 °C/min (comparatively fast) at T < 550 °C to ~5 °C/min (comparatively slow) at higher temperatures. The heating was arrested each time the release of hydrogen from BH_3NH_3 was expected or the growth of ternary Na–Si–H materials was detected. Pressure was estimated *in situ* from powder X-ray diffraction (PXRD) patterns using the equation of state (EOS) of NaCl by Matsui et al (Matsui et al., 2012). Temperature was evaluated from power – T calibration curves obtained by reproducing *in situ* runs offline using analogous 14/7 setups equipped with central C-type thermocouple. The effect of pressure on the thermocouple EMF in the studied range of p, T is expected to be negligible (Nishihara et al., 2020).

Angle-dispersive PXRD patterns were collected continuously in 1.27 – 15.26° 2θ range at a constant wavelength (λ = 0.233933 Å, E ≈ 53.0 keV), selected by the Si111 double-crystal monochromator from the emission of a U18 cryoundulator at ~6 mm magnetic gap. Data were acquired using the Pilatus3X-900 kW CdTe high-resolution 2D detector which was built and developed by DECTRIS specifically for the ID06-LVP beamline. Acquisition rate per dataset typically varied from every 60 sec during the compression to every second during the heating. Sample-to-detector distance and detector offset were calibrated using LaB_6-SRM660a (NIST). The *in situ* data were integrated, visualized and manipulated using the Fit2D software (Hammersley, 2016). Indexing of the powder patterns was performed using DICVOL and TAUP algorithms within the CRYSFIRE package (Shirley, 2004). For least-

squares refinement of unit cell parameters the UnitCell software was employed (Holland and Redfern, 1997).

2. Specific description of a 9 GPa run (2:1 NaH:Si composition)

The sample was heated upon reaching target pressure (~8.6 GPa). The plot showing temperature variations during the experiment is provided in Figure S1. At ~300 °C hydrogen release from NH_3BH_3 source was expected to be completed. During a ~12 min temperature dwell at ~380 °C a set of broad low intensity reflections appeared. The peaks could be indexed to a primitive hexagonal unit cell ($a \approx 4.75$ Å, $c \approx 7.65$ Å). However, this evaluation is rather approximate due to the weak intensity and diffuse nature of the reflections. Above ~560 °C the hexagonal phase was promptly replaced by another new set of reflections which were indexed to a primitive tetragonal unit cell (with a highest applicable space group being *P*4/*mbm* (№127). Shortly after the beginning of the growth (~610 °C) the lattice parameters of the tetragonal phase were estimated to $a \approx 6.49$ Å, $c \approx 4.73$ Å, while at highest temperature (~850 °C) they corresponded to $a \approx 6.59$ Å, $c \approx 4.78$ Å. Symmetry and cell parameters of the tetragonal phase strongly hinted at the isostructural relation to the double salts with K_3SiF_7-type structure, suggesting Na_3SiH_7 composition. Interestingly, even upon heating to 850 °C the growth of the tetragonal Na_3SiH_7 was very sluggish while noticeable amounts of unreacted NaH and Si were still present, which is attributed to a poor reactivity of elemental Si. Also, at this temperature the diffraction pattern of Na_3SiH_7 became noticeably textured. The sample was then slowly cooled (~5 °C/min) from ~850 °C to ~715 °C, and then at ~25 °C/min to RT. Upon cooling below 130 °C a (second-order) continuous phase transition of tetragonal Na_3SiH_7 to an orthorhombic *Pbam* polymorph was observed. The estimated pressure after cooling was ~6.2 GPa, and the cell parameters of the *Pbam* phase were approximated as: $a \approx 9.165$ Å, $b \approx 9.289$ Å, $c \approx 4.758$ Å.

The sample was decompressed over the course of ~12 hours. The phase transition of *Pbam*-Na_3SiH_7 to a yet unidentified low pressure (LP) polymorph started below ~4.5 GPa. The estimated cell parameters of *Pbam* phase at the onset of transformation were: $a \sim 9.29$ Å, $b \sim 9.42$ Å, $c \sim 4.81$ Å. Shortly after, at ~4.2 GPa, the pressure dropped rapidly to ~0.5 GPa, which obscured further characterization of LP phase. The sample was recovered at ambient conditions, however, its PXRD characterization was not conclusive. It is not clear whether Na_3SiH_7 is recoverable to ambient conditions.

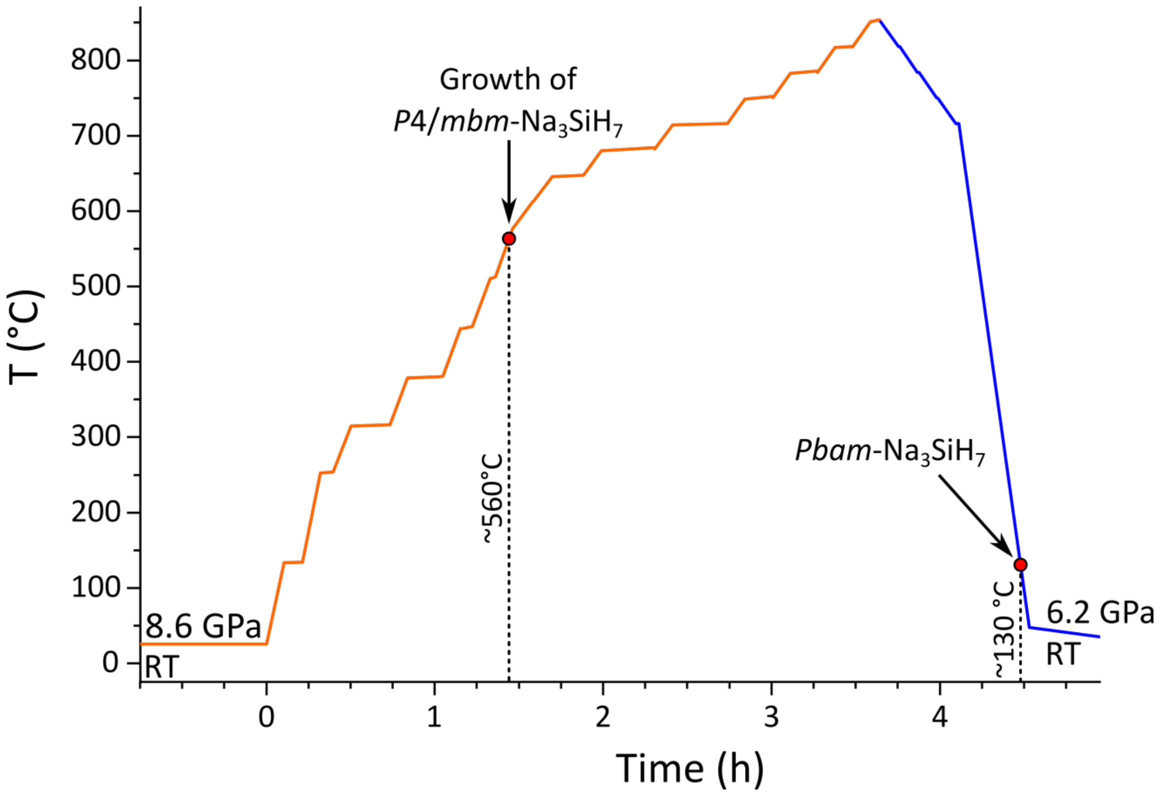

Figure S1. Temperature variations as a function of time for the NaH–Si–H_2 system at ≈9 GPa (2:1 NaH:Si ratio). Filled circles and dropped dashed lines mark the temperature and time points when the growth of tetragonal (*P*4/*mbm*) Na_3SiH_7 and its transition to the orthorhombic (*Pbam*) polymorph were detected. Orange and blue colors correspond heating and cooling, respectively.

3. Specific description of a 5 GPa run (2:1 NaH:Si composition)

The pressure before the start of the heating corresponded to ~5.1 GPa. The plot showing temperature variations during the experiment is provided in Figure S2. The sample was annealed at ~430 °C for approx. 20 min for equilibration after the hydrogen release. Afterwards the sample was heated to ~515 °C, and the heating was arrested for ~ 70 min. No growth of tetragonal Na_3SiH_7 was detected during this time. It was realized that at this point pressure had decreased to ~4.5 GPa. During the next 2 hours the pressure was increased to ~6.9 GPa (while keeping the power to the heater constant). The growth of tetragonal phase was seen at 510 °C above 5.2 GPa (cell parameters at the start of the growth were approx.: *a* ~ 6.65 Å, *c* ~ 4.83 Å). At ~6.9 GPa and ~490 °C its cell parameters corresponded (approximately) to *a* ~ 6.55 Å, *c* ~ 4.77 Å (the reflections were still very weak, which hindered more precise evaluation). Afterwards the temperature was carefully increased to ~840 °C while the Na_3SiH_7 growth proceeded very sluggishly, very similar to the 9 GPa experiment. At ~770 °C the cell parameters of tetragonal Na_3SiH_7 were refined to *a* = 6.59134(15) Å, *c* = 4.78027(15) Å. The weight percentage of Na_3SiH_7, NaH and Si at this point was estimated to be ~17.6 %, ~49.9% and ~32.5%, respectively.

From ~840 °C the sample was cooled at a rate of ~20 °C/min. The phase transition to *Pbam*-Na_3SiH_7 polymorph occurred below 120 °C. The pressure corresponded to ~5.2 GPa after cooling, and cell parameters of *Pbam* phase at this pressure were estimated to be $a \approx 9.227$ Å, $b \approx 9.357$ Å, $c \approx 4.757$ Å. The sample was decompressed over the course of ~9 hours. The transition to low pressure polymorph could be observed more clearly, as compared to the 9 GPa experiment. The onset of transformation started below ~4.3 GPa. Cell parameters of the *Pbam* phase at this point corresponded to $a \approx 9.28$ Å, $b \approx 9.41$ Å, $c \approx 4.79$ Å.

The intermediate hexagonal phase seen in the 9 GPa run was not observed at 5 GPa. However, the additional unidentified phase, growing in parallel with *P*4/*mbm*-Na_3SiH_7 (marked in Figure 3, main text) is also observed in the 5 GPa experiment, see Figure S4, and, as in the 9 GPa run, the compound decomposed above ~830 °C.

The 5 GPa run was reproduced using a sample of a different composition (1:1 NaH:Si molar ratio). However, changing ratio of the components did not have any significant effect on the reaction outcome or its kinetics. Instead of slow cooling the tetragonal Na_3SiH_7 was temperature-quenched which resulted in the instantaneous transformation to the *Pbam* phase.

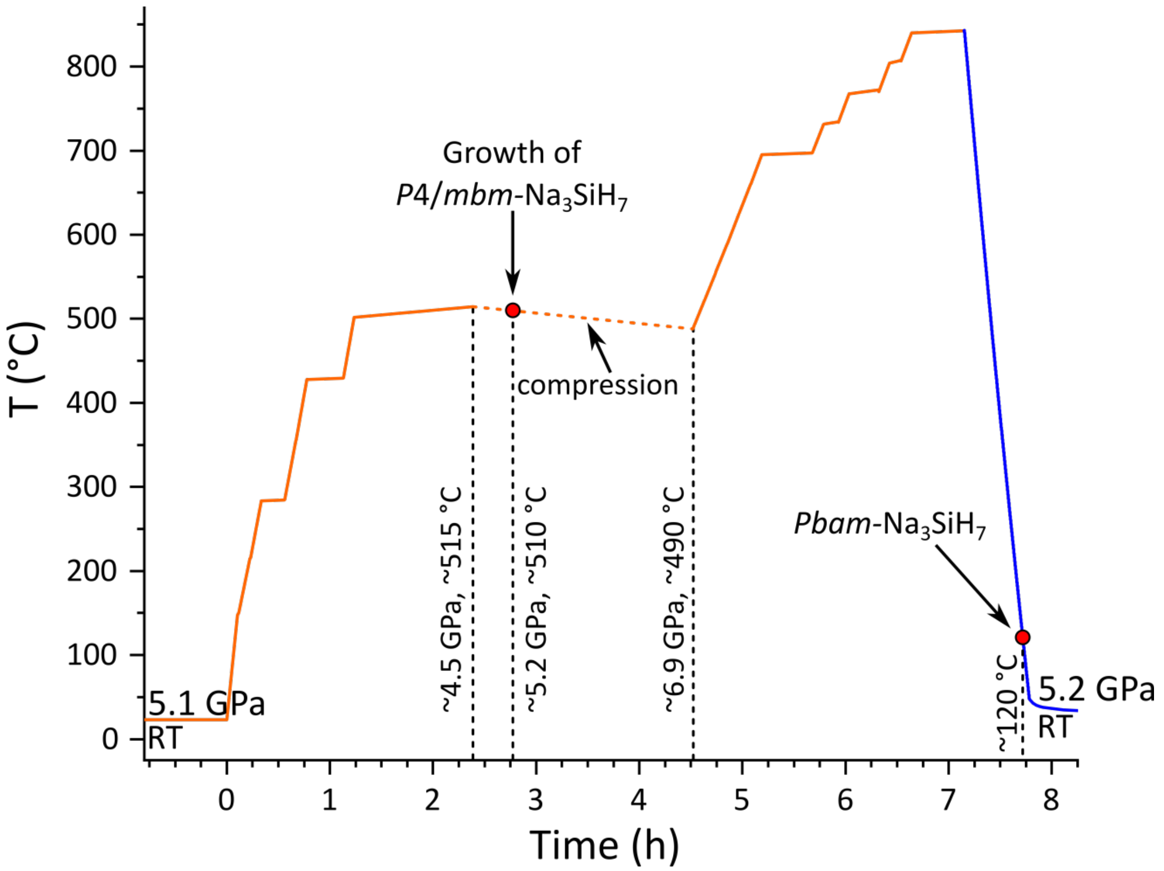

Figure S2. Temperature and pressure variations as a function of time for the NaH–Si–H_2 system in a 5 GPa run (2:1 NaH:Si ratio). Filled circles and dropped dashed lines mark the growth of tetragonal (*P*4/*mbm*) Na_3SiH_7 and its transition to orthorhombic (*Pbam*) polymorph. Orange and blue colors correspond heating and cooling, respectively.

4. Strucure characterization of P4/mbm-Na_3SiH_7

Le Bail fitting (Le Bail et al., 1988) and Rietveld refinement (Rietveld, 1969) against the *in situ* data were performed in Jana2006 (Petříček et al., 2014). PXRD patterns used for the analysis were acquired during the 5 GPa (NaH:Si=2:1) run after the second pressure increase (temperature dwell at ~770 C°, cf. Figure S2) where the pressure corresponded to ~7.2 GPa according to the EOS of NaCl (Matsui et al., 2012). At this point of the experiment the content of tetragonal Na_3SiH_7 sample was sufficient for fitting while the texture due to active recrystallization was not yet significant. The data were prepared for analysis by averaging ~130 sequential patterns in order to diminish possible effect of texture on the intensities as well as to improve signal-to-noise ratio. In addition, the observed intensities were corrected for background by subtracting the lowest non-zero value from the I_{obs} column. The structural model for the tetragonal Na_3SiH_7 used in the Rietveld refinement (SG №127, *P*4/*mbm*) corresponded to the calculated structure relaxed at 10 GPa (Table S5). The refined parameters included background (10th degree Chebyshev polynomials in combination with manually assigned points), zero shift, unit cell dimensions, peak profile parameters (corresponding to pseudo-Voigt function), scale factor, atomic coordinates for Na atoms at 4*h* Wyckoff site and ADPs for Na and Si atoms. The ADPs of Na atoms in Na_3SiH_7 structure were constrained together. The atomic coordinates and ADPs of the hydrogen atoms remained fixed during the refinement. The U_{iso} (H) were assigned an arbitrary value of 0.038 Å^2 (B_{iso} ≈ 3.0 Å^2). In addition, the following phases were included in the refinement: NaH (SG №225), Si (SG №227), NaCl (SG №225). Peaks originating from assembly materials, some of the NaCl peaks and the zero-intensity regions arising from the gaps between the detector chips were excluded from the refinement. Results of the Rietveld analysis for the tetragonal Na_3SiH_7 are shown in Tables S1 and S2 as well as in Figure S3.

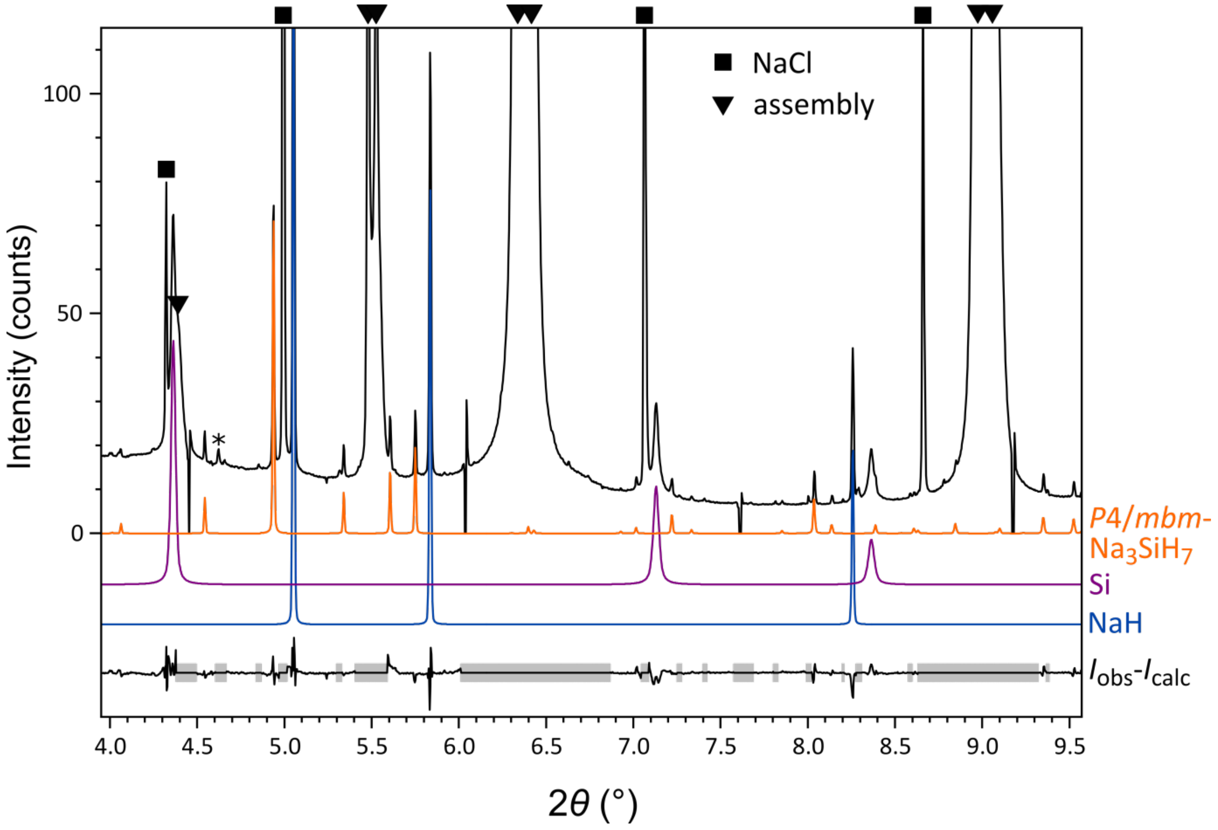

Figure S3. PXRD patterns of *P*4/*mbm*-Na_3SiH_7 (orange, magnified ×1.2), silicon (purple) and sodium hydride (blue) simulated from the results of Rietveld fit of the corresponding phases to the PXRD data (black) collected *in situ* at ID06-LVP, ESRF, during the 5 GPa run (2:1 NaH:Si starting mixture) upon temperature dwell at ~770 °C (λ = 0.233933 Å). Diffraction peaks corresponding to assembly (MgO), most of NaCl reflections, artifacts and zero-intensity regions arising from the gaps between the detector chips, and a weak peak of an unidentified impurity (marked with an asterisk) were excluded from the refinement. Excluded regions are marked by gray rectangles in the difference plot.

Table S1. Results of the Rietveld refinement of the *P*4/*mbm*-Na_3SiH_7 structure at ≈7.2 GPa, ≈770 °C.

Crystal system	Tetragonal
Space group	*P*4/*mbm* (127)
Z	2
Lattice parameters (Å)	*a* = 6.59134(15)<br>*c* = 4.78027(15)
V (Å^3)	207.683(9)
Formula weight (g/mol)	104.11
d_{calc} (g/cm^3)	1.6649
R_{obs} (%)	5.55
R_{all} (%)	10.72

Table S2. Fractional coordinates and atomic displacement parameters obtained for the *P4*/*mbm*-Na_3SiH_7 at ≈7.2 GPa, ≈770 °C. Hydrogen atom positions correspond to the DFT-optimized structure, see Table S5d. The coordinates and ADPs of H atoms remained fixed during the refinement.

**Atom**	***Wyck***	***x***	***y***	***z***	U_{iso} (Å^2)	B_{iso} (Å^2)
Na1	2*a*	0.0	0.0	0.0	0.058(4)	4.58(32)
Na2	4*h*	0.8024(7)	*x*+1/2	1/2	0.058(4)	4.58(32)
Si1	2*d*	0	1/2	0	0.047(5)	3.71(39)
H1	2*b*	0	0	1/2	0.038	3
H2	4*g*	0.8321	*x*+1/2	0	0.038	3
H3	8*k*	0.6166	*x*+1/2	0.2345	0.038	3

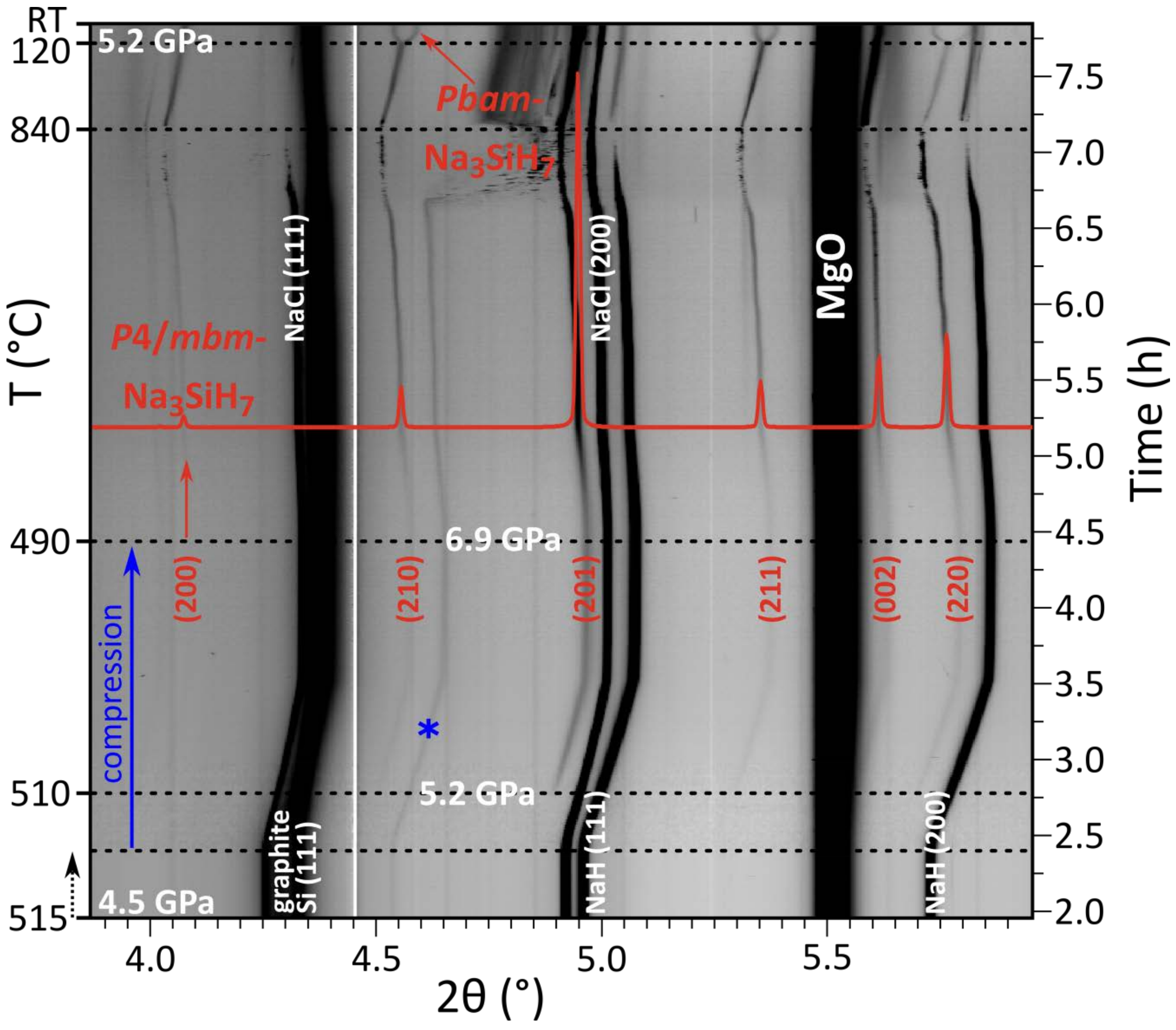

Figure S4. Compilation of PXRD patterns (λ = 0.233933 Å) acquired during the hydrogenation of 2NaH:1Si mixture in 5 GPa experiment (observed intensities are shown on logarithmic scale). Overlain is the simulated pattern of *P*4/*mbm*-Na_3SiH_7 at ≈700 °C (shown in red, linear scale) based on the refined model (Table S1-2). Splitting of its reflections upon transition to *Pbam* phase is visible on cooling below 120 °C. Peak of an additional unidentified phase growing in parallel with Na_3SiH_7 and disappearing above ≈830 °C is marked with a blue asterisk. This phase is also seen in the 9 GPa experiment, cf. Figure 3.

II. Theoretical calculations.

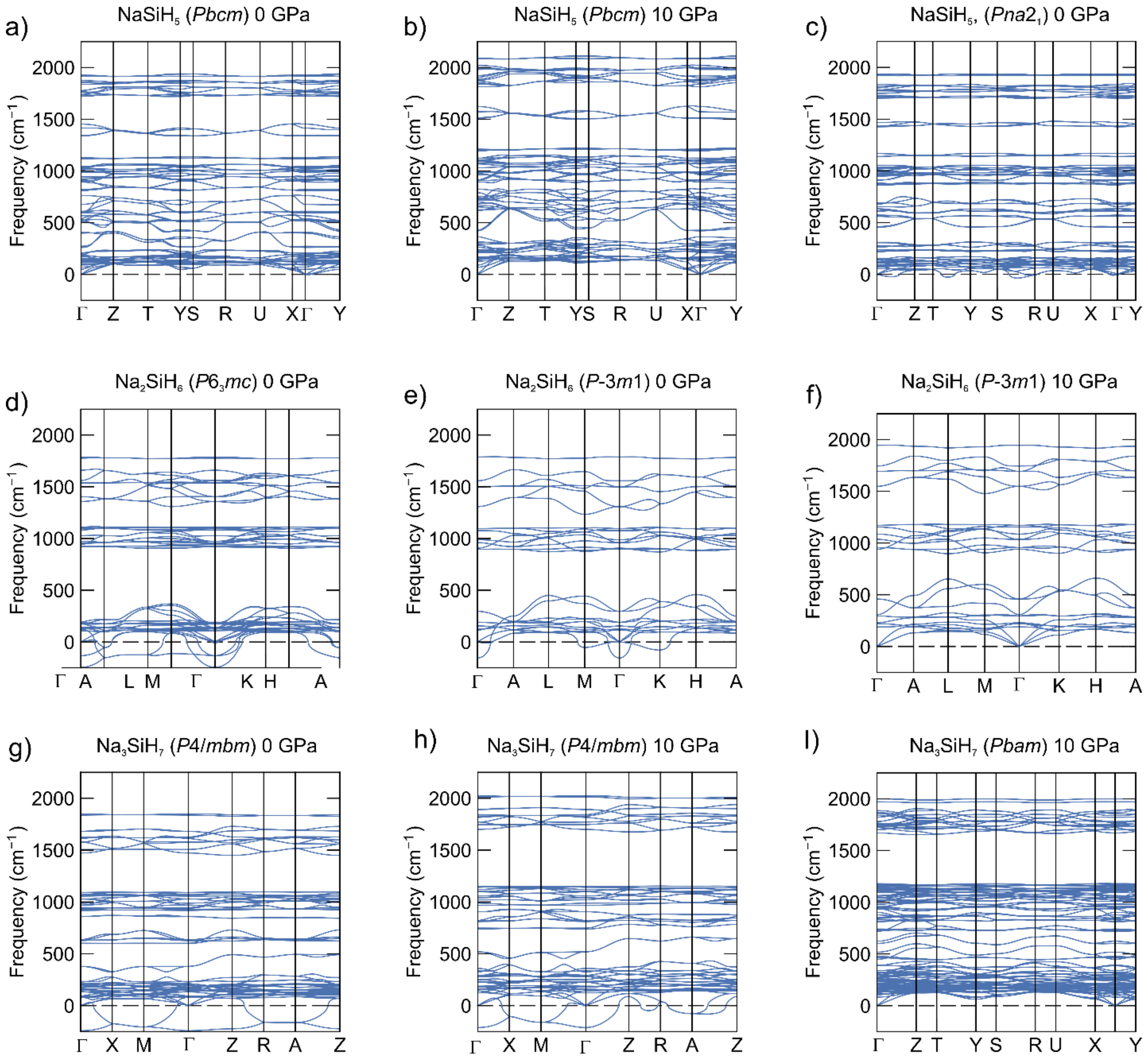

Figure S5. Phonon dispersions for $Na_mSiH_{(4+m)}$ compounds at 0 and 10 GPa (and zero Kelvin). At ambient pressure *Pbam* Na_3SiH_7 has a negative formation enthalpy (with respect to 3NaH + Si + $2H_2$) but is not dynamically stable (not shown). At ambient pressure Na_2SiH_6 (as $P6_3mc$) is slightly above the convex hull and, in addition, are not dynamically stable.

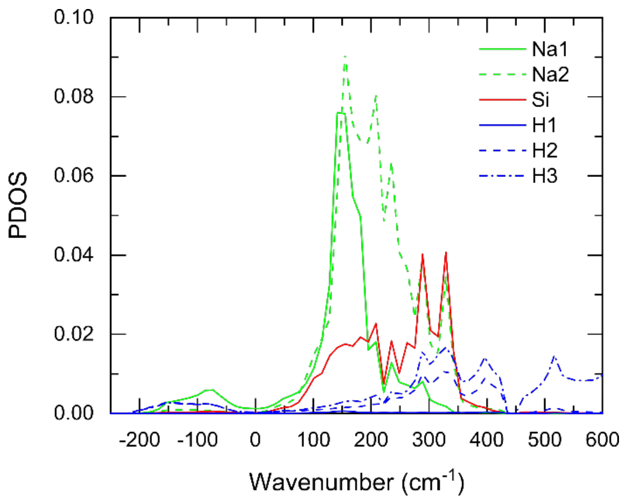

Figure S6. Phonon DOS (pDOS) of *P*4/*mbm*-Na_3SiH_7 at 10 GPa and zero Kelvin partitioned into atomic contributions of H, Na, and Si. (Na1 2*a* 0, 0, 0); Na2 4*h* 0.818, *x*+1/2, 1/2; Si 2*d* 0, ½, 0; H1 2*b* 0, 0, 1/2; H2 4*g* 0.822, *x*+1/2, 0; H3 8*k* 0.622, *x*+1/2, 0.243). Imaginary phonons are associated with Na2 and H2/H3 (which correspond to the H atoms of the SiH_6^{2-} unit)

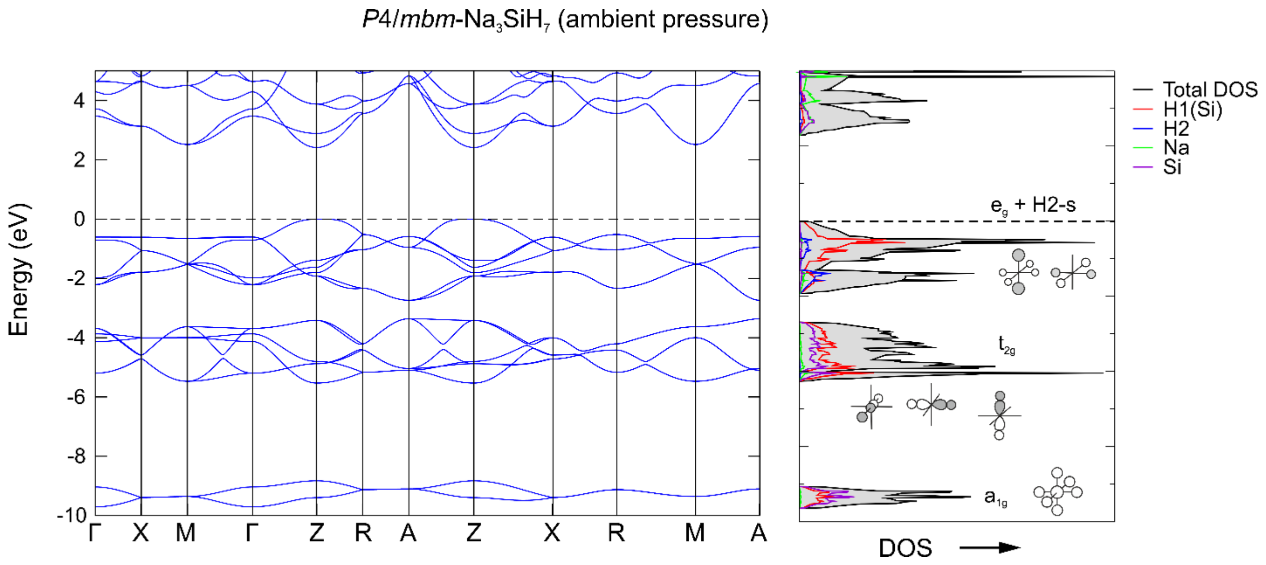

Figure S7: Electronic band structure (left) and density of states (DOS, right) of *P*4/*mbm*-Na_3SiH_7 at ambient pressure.

Table S3. Relaxed structures for *Pna*2_1 and *Pbcm* **$NaSiH_5$** at various pressures

a) *Pna*2_1 ($BaAlH_5$ structure), 0 GPa, dynamically unstable

a = 9.4343 Å, *b* = 6.4199 Å, *c* = 4.5638 Å

**Atom**	***Wyck***	***x***	***y***	***z***
Na1	4*a*	0.1678	0.6707	0.2607
Si1	4*a*	0.5574	0.3310	0.2276
H1	4*a*	0.4879	0.4459	-0.0918
H2	4*a*	0.0919	0.3648	0.0351
H3	4*a*	0.1184	0.2604	0.5141
H4	4*a*	-0.0999	0.2329	0.2847
H5	4*a*	0.1938	0.0469	0.1366

b) *Pbcm* ($SrAlH_5$ structure), 10 GPa, stable

a = 3.9831 Å, *b* = 11.1837 Å, *c* = 4.6070 Å

**Atom**	***Wyck***	***x***	***y***	***z***
Na1	4*d*	0.1599	0.0940	1/4
Si1	4*d*	0.4236	0.8433	1/4
H1	4*c*	0.3997	0.25	0
H2	4*d*	0.2247	0.4010	1/4
H3	4*d*	0.8735	0.2591	1/4
H4	8*e*	0.2999	0.5761	-0.0013

c) *Pbcm* ($SrAlH_5$ structure), 20 GPa, stable

a = 3.7831 Å, b = 10.7567 Å, c = 4.4374 Å

**Atom**	***Wyck***	***x***	***y***	***z***
Na1	4*d*	0.1430	0.0880	1/4
Si1	4*d*	0.4533	0.8424	1/4
H1	4*c*	0.3454	1/4	0
H2	4*d*	0.1810	0.4062	1/4
H3	4*d*	0.8504	0.2533	1/4
H4	8*e*	0.3253	0.5765	0.0080

Table S4. Relaxed structures for *P*6$_3$mc and *P*$\bar{3}$*m*1 **Na_2SiH_6** at various pressures

a) *P*$\bar{3}$*m*1, 0 GPa, dynamically unstable

a = 4.9344 Å, *c* = 4.1271 Å

**Atom**	***Wyck***	***x***	***y***	***z***
Na1	2d	1/3	2/3	0.2692
Si1	1a	0	0	0
H1	6i	0.1550	-*x*	0.7770

b) *P*$\bar{3}$*m*1, 10 GPa

a = 4.6239 Å, *c* = 3.7548 Å

**Atom**	***Wyck***	***x***	***y***	***z***
Na1	2*d*	1/3	2/3	0.2753
Si1	1*a*	0	0	0
H1	6*i*	0.1632	-*x*	0.7623

c) *P*6$_3$*mc*, 0 GPa, dynamically unstable

a = 5.0047 Å, *c* = 8.1448 Å

**Atom**	***Wyck***	***x***	***y***	***z***
Na1	2b	1/3	2/3	0.8674
Na2	2a	0	0	0.6060
Si1	2b	1/3	2/3	0.2480
H1	6c	0.5160	-*x*	0.6329
H2	6c	0.1797	-*x*	0.3607

d) $P6_3mc$, 0 GPa, dynamically unstable

a = 5.0047 Å, c = 8.1448 Å

**Atom**	***Wyck***	***x***	***y***	***z***
Na1	2b	1/3	2/3	0.8674
Na2	2a	0	0	0.6060
Si1	2b	1/3	2/3	0.2480
H1	6c	0.5160	-*x*	0.6329
H2	6c	0.1797	-*x*	0.3607

Table S5. Relaxed structures for *P*4/*mbm* and *Pbam* Na_3SiH_7 at various pressures

a) *Pbam*, 0 GPa, dynamically unstable

a = 9.5385 Å, *b* = 9.9515 Å, *c* = 4.9580 Å

**Atom**	***Wyck***	***x***	***y***	***z***
Na1	4*g*	0.2520	0.2803	0
Na2	4*h*	0.4897	0.6958	1/2
Na3	4*h*	0.6923	-0.0151	1/2
Si1	2*c*	0	1/2	0
Si2	2*a*	0	0	0
H1	4*h*	0.2649	0.2375	1/2
H2	4*g*	0.4567	0.6564	0
H3	4*g*	0.6535	-0.0649	0
H4	8*i*	0.4527	0.8945	0.2296
H5	8*i*	0.8854	0.0307	0.7703

b) *Pbam*, 5 GPa, borderline dynamically unstable

a = 9.1114 Å, *b* = 9.3881 Å, *c* = 4.7574 Å

**Atom**	***Wyck***	***x***	***y***	***z***
Na1	4*g*	0.2538	0.2810	0
Na2	4*h*	0.4883	0.6890	1/2
Na3	4*h*	0.6866	-0.0169	1/2
Si1	2*c*	0	1/2	0
Si2	2*a*	0	0	0
H1	4*h*	0.2640	0.2460	1/2
H2	4*g*	0.4700	0.6675	0
H3	4*g*	0.6645	-0.0567	0
H4	8*i*	0.4601	0.8869	0.2367

H5	8*i*	0.8799	0.0214	0.7623

c) *Pbam*, 10 GPa, dynamically stable

a = 8.8368 Å, *b* = 9.0550 Å, *c* = 4.6351 Å

**Atom**	***Wyck***	***x***	***y***	***z***
Na1	4*g*	0.2538	0.2807	0
Na2	4*h*	0.4884	0.6863	1/2
Na3	4*h*	0.6843	-0.0180	1/2
Si1	2*c*	0	1/2	0
Si2	2*a*	0	0	0
H1	4*h*	0.2627	0.2500	1/2
H2	4*g*	0.4751	0.6730	0
H3	4*g*	0.6701	-0.0532	0
H4	8*i*	0.4631	0.8830	0.2410
H5	8*i*	0.8772	0.0181	0.7577

d) *P*4/*mbm*, 0 GPa, dynamically unstable

a = 6.7781 Å, *c* = 4.8794 Å

**Atom**	***Wyck***	***x***	***y***	***z***
Na1	2*a*	0	0	0
Na2	4*h*	0.8166	*x*+1/2	1/2
Si1	2*d*	0	1/2	0
H1	2*b*	0	0	1/2
H2	4*g*	0.8321	*x*+1/2	0
H3	8*k*	0.6166	*x*+1/2	0.2345

e) *P*4/*mbm*, 10 GPa, dynamically unstable

a = 6.2754 Å, *c* = 4.6250 Å

**Atom**	***Wyck***	***x***	***y***	***z***
Na1	2*a*	0	0	0
Na2	4*h*	0.8185	*x*+1/2	0.5
Si1	2*d*	0	1/2	0
H1	2*b*	0	0	0.5
H2	4*g*	0.8225	*x*+1/2	0
H3	8*k*	0.6228	*x*+1/2	0.2437

References

Guignard, J., and Crichton, W. A. (2015). The large volume press facility at ID06 beamline of the European synchrotron radiation facility as a High Pressure-High Temperature deformation apparatus. *Rev. Sci. Instrum.* 86, 085112. doi: 10.1063/1.4928151.

Hammersley, A. P. (2016). *FIT2D*: a multi-purpose data reduction, analysis and visualization program. *J. Appl. Crystallogr.* 49, 646–652. doi: 10.1107/S1600576716000455.

Holland, T. J. B., and Redfern, S. A. T. (1997). Unit cell refinement from powder diffraction data: the use of regression diagnostics. *Mineralogical Magazine* 61, 65–77. doi: 10.1180/minmag.1997.061.404.07.

Le Bail, A., Duroy, H., and Fourquet, J. L. (1988). Ab-initio structure determination of $LiSbWO_6$ by X-ray powder diffraction. *Mater. Res. Bull.* 23, 447–452. doi: 10.1016/0025-5408(88)90019-0.

Matsui, M., Higo, Y., Okamoto, Y., Irifune, T., and Funakoshi, K.-I. (2012). Simultaneous sound velocity and density measurements of NaCl at high temperatures and pressures: Application as a primary pressure standard. *Am. Mineral.* 97, 1670–1675. doi: 10.2138/am.2012.4136.

Nishihara, Y., Doi, S., Kakizawa, S., Higo, Y., and Tange, Y. (2020). Effect of pressure on temperature measurements using WRe thermocouple and its geophysical impact. *Phys. Earth Planet. Inter.* 298, 106348. doi: 10.1016/j.pepi.2019.106348.

Nylén, J., Sato, T., Soignard, E., Yarger, J. L., Stoyanov, E., and Häussermann, U. (2009). Thermal decomposition of ammonia borane at high pressures. *J. Chem. Phys.* 131, 104506. doi: 10.1063/1.3230973.

Petříček, V., Dušek, M., and Palatinus, L. (2014). Crystallographic Computing System JANA2006: General features. *Z. Kristallogr. - Cryst. Mater.* 229, 345–352. doi: 10.1515/zkri-2014-1737.

Rietveld, H. M. (1969). A profile refinement method for nuclear and magnetic structures. *J. Appl. Crystallogr.* 2, 65–71. doi: 10.1107/S0021889869006558.

Shirley, R. (2004). Crysfire 2004: An interactive powder indexing support system. 41 Guildford Park Avenue, Guildford, Surrey, UK, 2004.

Vekilova, O. Yu., Beyer, D. C., Bhat, S., Farla, R., Baran, V., Simak, S. I., et al. (2023). Formation and Polymorphism of Semiconducting K_2SiH_6 and Strategy for Metallization. *Inorg. Chem.* 62, 8093–8100. doi: 10.1021/acs.inorgchem.2c04370.
